# Miniaccess open repair of descending thoracic aorta

**DOI:** 10.1016/j.xjtc.2021.03.031

**Published:** 2021-04-09

**Authors:** Younju Rhee, Joon Bum Kim

**Affiliations:** a Department of Thoracic and Cardiovascular Surgery, Chungnam National University Hospital, Chungnam National University School of Medicine, Daejeon, South Korea; b Department of Thoracic and Cardiovascular Surgery, Asan Medical Center, University of Ulsan College of Medicine, Seoul, South Korea

**Figure:**
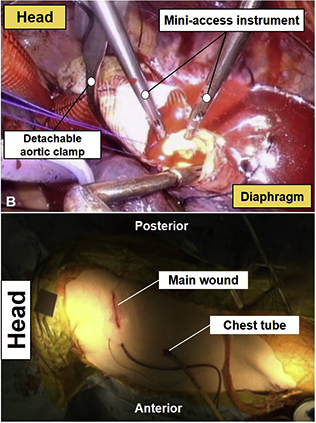
Miniaccess open repair of descending thoracic aorta through a limited skin incision.

**Central Message**

The miniaccess open repair of descending thoracic aorta showed favorable early outcomes and acceptable safety in selected patients.

See Commentary on page 31.

Thoracic endovascular repair (TEVAR) has emerged as an effective treatment option for descending thoracic aorta (DTA) diseases due to less invasiveness and better early outcomes compared with open repair. Nevertheless, potential endoleak, risks of later reintervention, and less effectiveness in challenging aortic anatomy are major limitations of TEVAR, and these still leave questions regarding its long-term durability.^1^, ^2^, ^3^ For these reasons, open DTA repair remains the most effective treatment option for DTA diseases, and its surgical outcomes have been improving with advances in surgical techniques and perioperative management. Open DTA repair requires extended thoracotomy involving a large skin incision with division of thoracic muscles and cutting costal cartilage/bone, which in turn carries extensive surgical trauma and its associated complications. To overcome these limitations, we have adopted miniaccess open DTA repair. Herein, we present our experiences and early outcomes of this procedure.

## Methods

Miniaccess open DTA repair is defined as limited skin incision (6-10 cm), sparing 2 thoracic muscles (ie, latissimus dorsi and serratus anterior muscles), saving costal cartilage/bone without cutting, and utilization of thoracoscopy and miniaccess instruments. All cases requiring elective aortic intervention on DTA were considered for this miniaccess surgery if the longitudinal extent of the lesion was within 15 cm in the aim of offering more durable repair with minimum surgical trauma. Exclusion criteria were emergent cases and high estimated surgical risks (ie, severe lung disease or chronic kidney disease) in which the conventional open thoracotomy or TEVAR were the preferable option.

**Figure 1:**
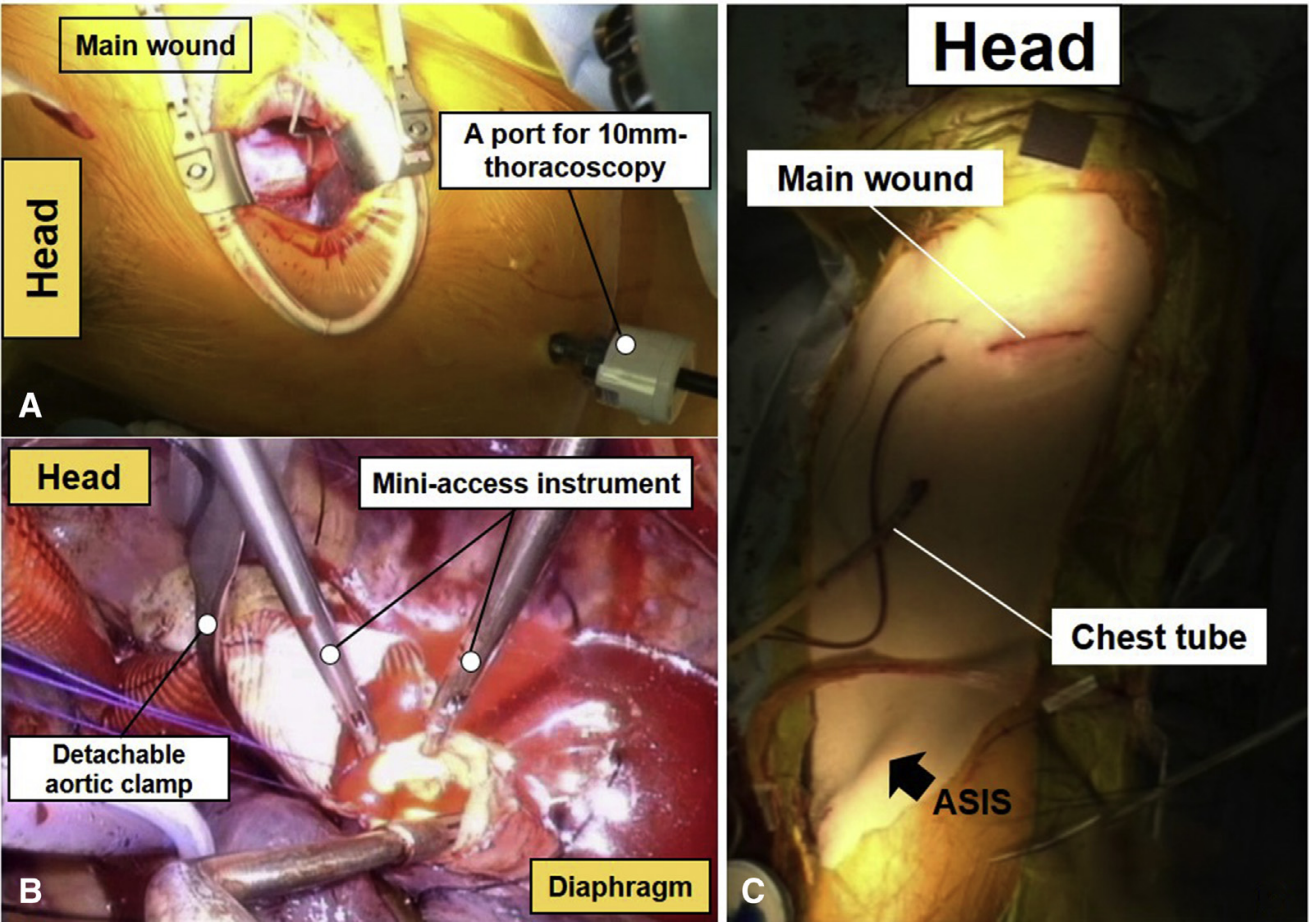
Miniaccess descending thoracic aorta open repair. A, The operation view is obtained either via direct vision through the main incision or thoracoscopy. B, Operation view in the process of distal anastomosis using miniaccess instruments obtained by thoracoscopy. C, Postoperative picture presenting a limited main incision and a chest tube inserted through the 10-mm port. *ASIS*, Anterior superior iliac spine.

Cerebrospinal fluid drainage was not conducted in all patients because the extent of the lesion was limited to involving the upper or middle DTA, and patients were relatively young with low-risk profiles at baselines. The patient was positioned on a bean bag for right lateral decubitus position similar to the position for conventional DTA or thoracoabdominal aortic open repair, but with the left shoulder pulled upward only (not anteriorly) because the main incision does not require lifting up the scapula anteriorly. A skin incision was made and 2 thoracic muscles were divided and spared along the planes without cutting. The access was made through the fourth or fifth intercostal space. A port for 10-mm thoracoscopy was inserted onto the sixth or seventh intercostal space, which were 2 levels lower than the main incision. With the operation view obtained either via direct vision or thoracoscopy, DTA was dissected from the surrounding structures with miniaccess instruments (Figure 1, *A* and *B*). Cardiopulmonary bypass was established through left femoral vessels under normothermic condition; however, moderate hypothermic circulatory arrest was used (nasopharyngeal temperature, 28°C) in cases where open distal arch repair was required. The aortic crossclamping was made at the proximal and distal segments of the target lesion using the detachable CardioVision MIC-Aortic Glauber Clamp (Cardiomedical GmbH, Langenhagen, Germany). After making a longitudinal incision of the target lesion, intercostal arteries were occluded by suture ligation or by vascular clips. Proximal and distal anastomoses were made by continuous running suture with 3–0 polypropylene, and multiple pledgetted mattress sutures were routinely added around the anastomotic lines for hemostatic reinforcement. At completion of the surgery, a chest tube was inserted through the port site (Figure 1, *C*, Video 1).

Early death and complications were defined as those occurring within 30 days postoperatively or during index hospitalization. The study was approved by the institutional ethics committee/review board (study number 2020-0630), and the requirement for informed patient consent was waived in view of the retrospective nature of the study.

## Results

From November 2018 through February 2020, 9 patients received miniaccess open repair of DTA aneurysm by a single surgeon in our center. Baseline characteristics and operative profiles are detailed in Tables 1 and 2, respectively. Two patients required postoperative blood product transfusion (1 U packed red blood cells in each) and the pain numeric rating scale for surgical site pain showed median of 1 (interquartile range, 1-2 [maximum score, 10]) on discharge day. There were no cases of early death, neurologic injuries, re-exploration due to bleeding, wound or vascular complications, or prolonged mechanical ventilator support (ie, >48 hours). Chylothorax and vocal cord injury occurred in 1 patient each (Table 3).

**Table 1:** Baseline characteristics of the patient population (N = 9)

Characteristic	Result
Age (y)	63 (range, 30-73; IQR, 51-70)
Male sex	6 (66.7)
Hypertension	5 (55.6)
Diabetes mellitus	2 (22.2)
Chronic kidney disease	0
Chronic obstructive pulmonary disease	0
Cancer within 5 y	1 (11.1)
Peripheral arterial obstructive disease	0
Coronary artery disease requiring intervention	1
Prior aortic surgery	2 (22.2)
Prior descending thoracic aortic surgery	0
Etiology	
Degenerative	7 (77.8)
Mycotic	1 (11.1)
Traumatic	1 (11.1)
EuroSCORE II	1.44 (range, 0.95-6.2; IQR, 0.95-1.85)

Values are presented as n (%) or median (range; interquartile range [IQR]). *EuroSCORE II*, European System for Cardiac Operative Risk Evaluation II.

**Table 2:** Operative profiles of the patient population (N = 9)

Profile	Result
Type of mechanical circulatory support	
Cardiopulmonary bypass	8 (88.9)
ECMO	1 (11.1)
Concomitant distal arch repair	2 (22.2)
Revascularization of segmental arteries	1 (11.1)
Procedure time	
Cardiopulmonary bypass time (min)	79 (range, 55-123; IQR, 67-84)
Entire procedure time (min)	239 (range, 195-314; IQR 212-254)

Variables are n (%) or median (range; interquartile range [IQR]). *ECMO,* Extracorporeal membrane oxygenation.

**Table 3:** Early outcomes of the patient population (N = 9)

Outcome	Result
Early death	0
Postoperative complications	
Stroke	0
Paraplegia or paraparesis	0
Re-exploration for bleeding control	0
Prolonged ventilator support (>48 h)	0
Postoperative pneumonia	0
Chylothorax	1 (11.1)
Vocal cord injury	1 (11.1)
Postoperative transfusion∗	2 (22.2)
Pain numeric rating scale (from 0 to 10 being maximum)	
Maximum scale to postoperative day 2	3 (range, 3-6; IQR, 3-6)
Maximum from postoperative day 3 to 5	4 (range, 2-6; IQR, 3-5)
Scale on discharge day	1 (range, 0-3; IQR, 1-2)
Drainage amount of chest tube (mL/d)	318 (range, 260-678; IQR, 300-360)
Duration of chest tube drainage (d)	5 (range, 3-21†; IQR, 4-9)
Length of stay (d)	
Intensive care unit stay	1 (range, 1-2; IQR, 1-2)
Postoperative hospitalization stay	7 (range, 5-22†; IQR, 6-11)

Values are presented as n (%) or median (range; interquartile range [IQR]).

∗ Each patient required 1 U packed red blood cells.

† A patient with chylothorax had a chest tube up to postoperative day 21, and except for this patient, the longest chest tube maintenance period was 10 days. The patient with chylothorax was discharged the day after the chest tube was removed.

**Figure 2:**
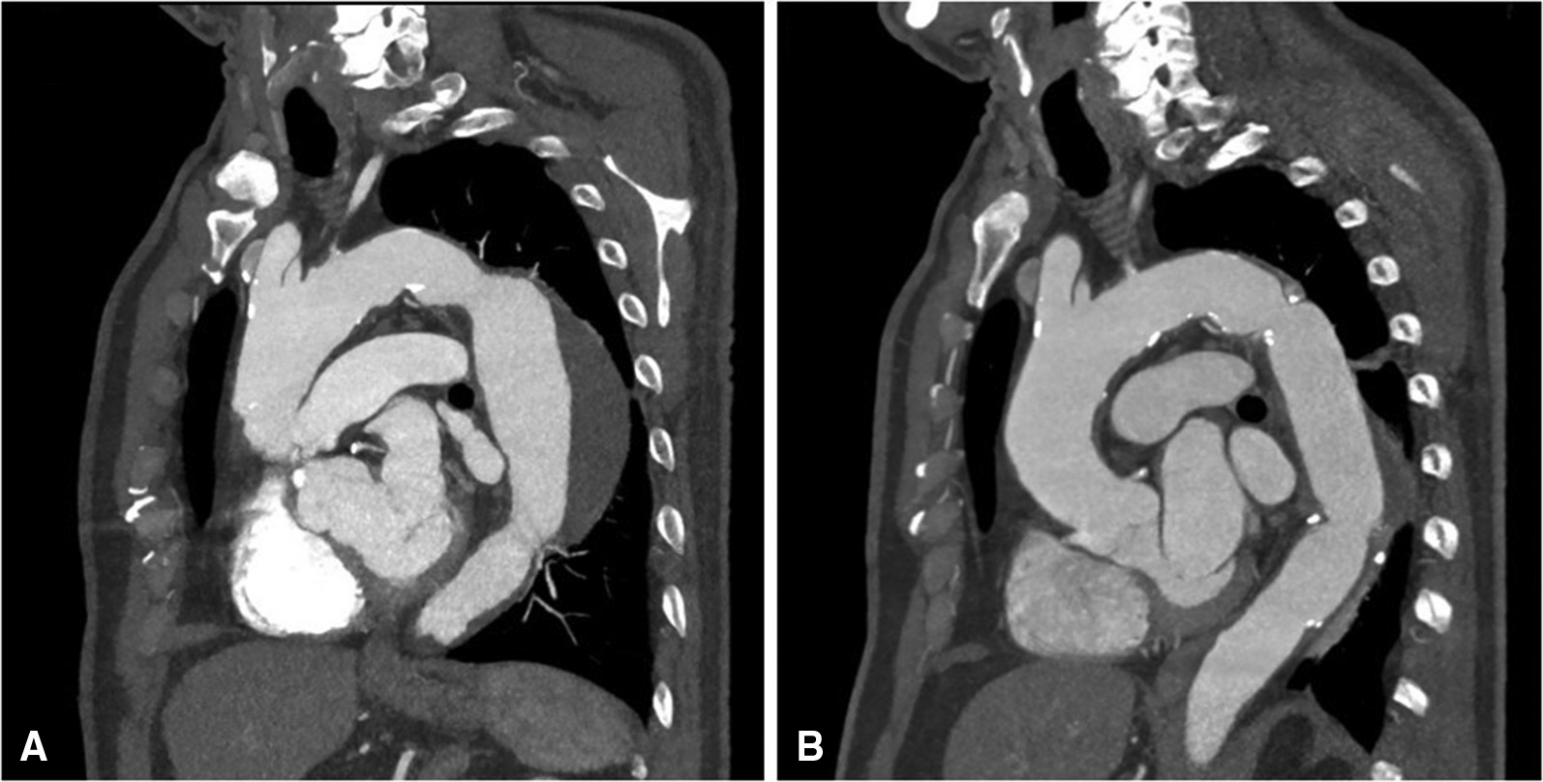
Preoperative (A) and postoperative computed tomography (CT) scans at 6 months (B) of a patient with degenerative aneurysm of 64 mm in diameter and 90 mm in length. Postoperative CT scan is routinely performed immediately after surgery during the index hospitalization and 3 to 12 months after surgery in all patients.

The follow-up duration was complete in all patients with a median duration of 11 months (range, 6-21 months). During the follow-up period, there were no additional complications, including postthoracotomy neuralgia. The patient who experienced vocal cord injury achieved complete resolution 3 months after surgery. Postoperative computed tomography was performed at 3 to 12 months after surgery in all patients, and there were no abnormal findings in all (Figure 2).

## Discussion

Even in the era of minimally invasive cardiac surgery, such approaches have not been widely utilized in open DTA repair because of the challenging nature of the procedures and their perceived high risks of perioperative morbidity and mortality.^1^^,^^3^ Open DTA repair requires a comprehensive understanding of the anatomy and delicate control throughout the entire procedure, for which a fully exposed surgical field has been regarded essential for safe execution of the surgery. For this, extended thoracotomy involving large skin incision and cutting of the thoracic muscles/ribs has been regarded a requisite to achieve optimal surgical field. This inevitably carries extensive surgical trauma and consequent postoperative pain, which is associated with impaired pulmonary functions and oftentimes with postoperative pneumonia. TEVAR is an excellent alternative to circumvent these problems; however, it also has its inherent limitations such as risks of endoleak and stent-related injuries. Miniaccess open DTA repair may be an excellent option to overcome the limitations of these conventional open repair and TEVAR approaches; however, there have been only few studies on utilizing miniaccess to address thoracic aortic diseases.^4^^,^^5^

To alleviate extensive surgical trauma, we have adopted miniaccess open DTA repair to obtain enough surgical exposure in a limited skin incision, thoracoscopic guidance was also utilized. Even with rigorous efforts to maximize exposure, we speculated that there would be a certain limiting longitudinal length in DTA segments that could be covered by this approach. By this logic, we selected cases prudently with aortic lesions within 10 to 15 cm in length. As a result, our preliminary experiences demonstrated the excellent early outcomes shown in this article. Patients with connective tissue disease can also be subject to this procedure if they meet this criterion (ie, extent length <15 cm); however, the application will be limited because those patients usually present with extensive aortic lesions.

## Conclusions

Our early experiences with miniaccess open DTA repair showed favorable early outcomes and acceptable safety in selected patients, which necessitate validations from further studies in larger cohorts.

## Supplementary Data

Video 1 A 47-year-old male patient with rapidly progressing mycotic aneurysm in descending thoracic aorta received miniaccess open aortic repair. The detailed step-by-step procedures are demonstrated. Video available at: https://www.jtcvs.org/article/S2666-2507(21)00283-2/fulltext.
