# Supplementary figures and images for: Miniaccess open repair of descending thoracic aorta
Source: JTCVS Tech. 2021 Apr 9;8:27–30. doi: 10.1016/j.xjtc.2021.03.031 (PMC8350782; doi:10.1016/j.xjtc.2021.03.031)

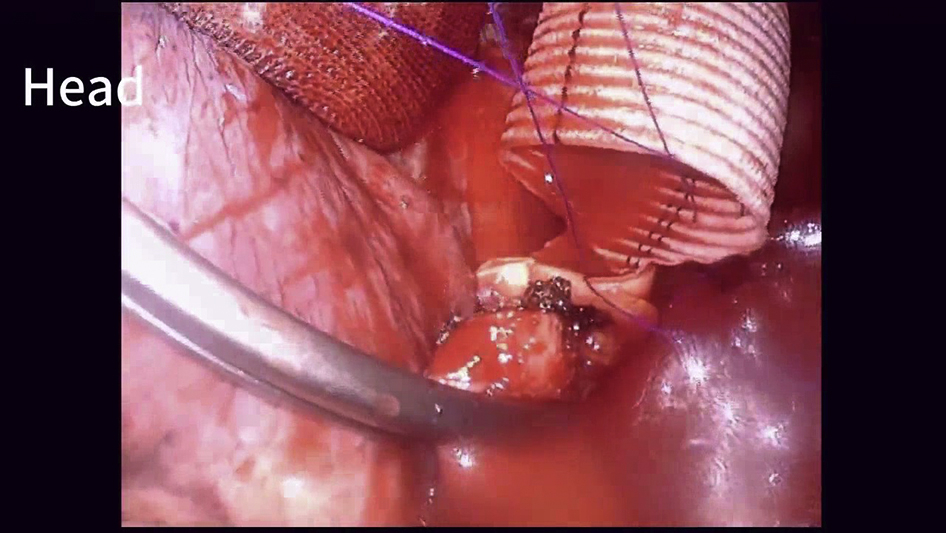

Supplement: Video 1 — A 47-year-old male patient with rapidly progressing mycotic aneurysm in descending thoracic aorta received miniaccess open aortic repair. The detailed step-by-step procedures are demonstrated. Video available at: https://www.jtcvs.org/article/S2666-2507(21)00283-2/fulltext. [file fx2.jpg]
